# Booly: a new data integration platform

**DOI:** 10.1186/1471-2105-11-513

**Published:** 2010-10-13

**Authors:** Long H Do, Francisco F Esteves, Harvey J Karten, Ethan Bier

**Affiliations:** 1 Section of Cell and Developmental Biology, University of California, San Diego, 9500 Gilman Drive, La Jolla, CA 92093-0349, USA; 2 Departments of Neurosciences and Psychiatry, School of Medicine, University of California, San Diego, La Jolla, California 92093-0608, USA

## Abstract

**Background:**

Data integration is an escalating problem in bioinformatics. We have developed a web tool and warehousing system, Booly, that features a simple yet flexible data model coupled with the ability to perform powerful comparative analysis, including the use of Boolean logic to merge datasets together, and an integrated aliasing system to decipher differing names of the same gene or protein. Furthermore, Booly features a collaborative sharing system and a public repository so that users can retrieve new datasets while contributors can easily disseminate new content.

**Results:**

We illustrate the uses of Booly with several examples including: the versatile creation of homebrew datasets, the integration of heterogeneous data to identify genes useful for comparing avian and mammalian brain architecture, and generation of a list of Food and Drug Administration (FDA) approved drugs with possible alternative disease targets.

**Conclusions:**

The Booly paradigm for data storage and analysis should facilitate integration between disparate biological and medical fields and result in novel discoveries that can then be validated experimentally. Booly can be accessed at http://booly.ucsd.edu.

## Background

The difficulty in achieving lasting solutions to integration of diverse biological data continues to be a central problem in bioinformatics [1,2]. A number of technologies and systems have been developed that offer a variety of potential solutions to the data integration problem. These solutions differ by the architecture they adopt (data warehousing [BioMART, BioWarehouse], link integration [SRS, Entrez, TAMBIS, BioZon], semantic integration [BioMoby, Bio2RDF]) and by the common "touch-points" used to integrate data (e.g., data values, names, identities, schema properties, ontology terms, Uniform Resource Identifier [URI], keywords, loci, spatial-temporal points) [1,3-11].

One major hurdle in current data integration efforts is the issue of naming and identity such that a variety of aliases exist for many of the same genes, proteins, and keywords. For example, Goble et al. states: "The failure to address identity will be the most likely obstacle that will stop mashups, or any other technology or strategy, becoming an effective integration mechanism." Another shortcoming of current data integration architectures is the high barrier of entry for contributions from both developers and the general research public. The data integration systems currently in existence either do not account for the general researcher contribution or are too difficult to utilize by non-specialists.

We have developed a data integration platform with an easy to use web interface that allows a broad variety of users to perform powerful comparative analysis between disparate datasets, including the use of Boolean operations (union, intersection, subtraction) and concatenated comparisons. An important element of this system is an integrated streamlined aliasing system to address the identifier problem by deciphering differing names of the same gene or protein. Furthermore, we have created a simple yet flexible data model so that the barrier of entry for both developers and researchers is minimal. For example, researchers can easily create new datasets by cutting and pasting their data from spreadsheet tables, while developers will have direct access through our Representational State Transfer (REST) style web services. Finally, we have created a collaborative sharing system and a repository within Booly so that users can retrieve new datasets while contributors can easily and securely share new content, allowing for fast creation and dissemination of entirely new databases with minimal effort.

We anticipate that the novel framework Booly provides for storing and integrating biological databases, with contributions from general researchers and large data centers alike coupled with high-throughput on demand alias translations, will spark a new approach in data integration efforts. These advantages over existing data integration approaches should attract growing contributions from developers and the research community that spur important new discoveries.

## Overview

**Figure 1:**
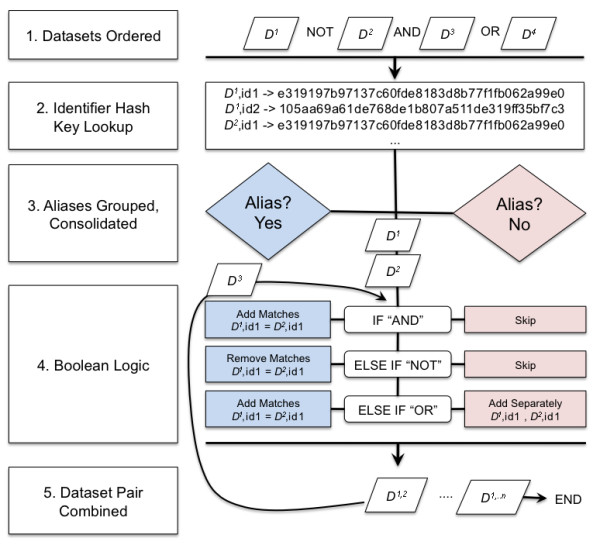
**The Booly data integration algorithm**. Overview of the steps involved in performing a Booly query.

The Booly data integration platform consists of a data warehouse, scripts to perform alias lookups and Boolean operations, and a web interface for interaction from the user. In Booly, data from Gene Ontology [12] and PubMed are represented as individual datasets similar to a spreadsheet table consisting of rows and columns. Each dataset can be merged with others to produce an output of the requested combination of Boolean operations constrained against the identifiers and their aliases grouped by a similar fingerprint such as gene sequence or chemical formula (Methods, Figure 1). For example, one can merge a table of microarray data with a Gene Ontology dataset to attach annotation to previously unannotated microarray data. Furthermore, heterogeneous identifiers from the datasets are resolved by the integrated alias lookups and applied accordingly.

### Implementation

**Figure 2:**
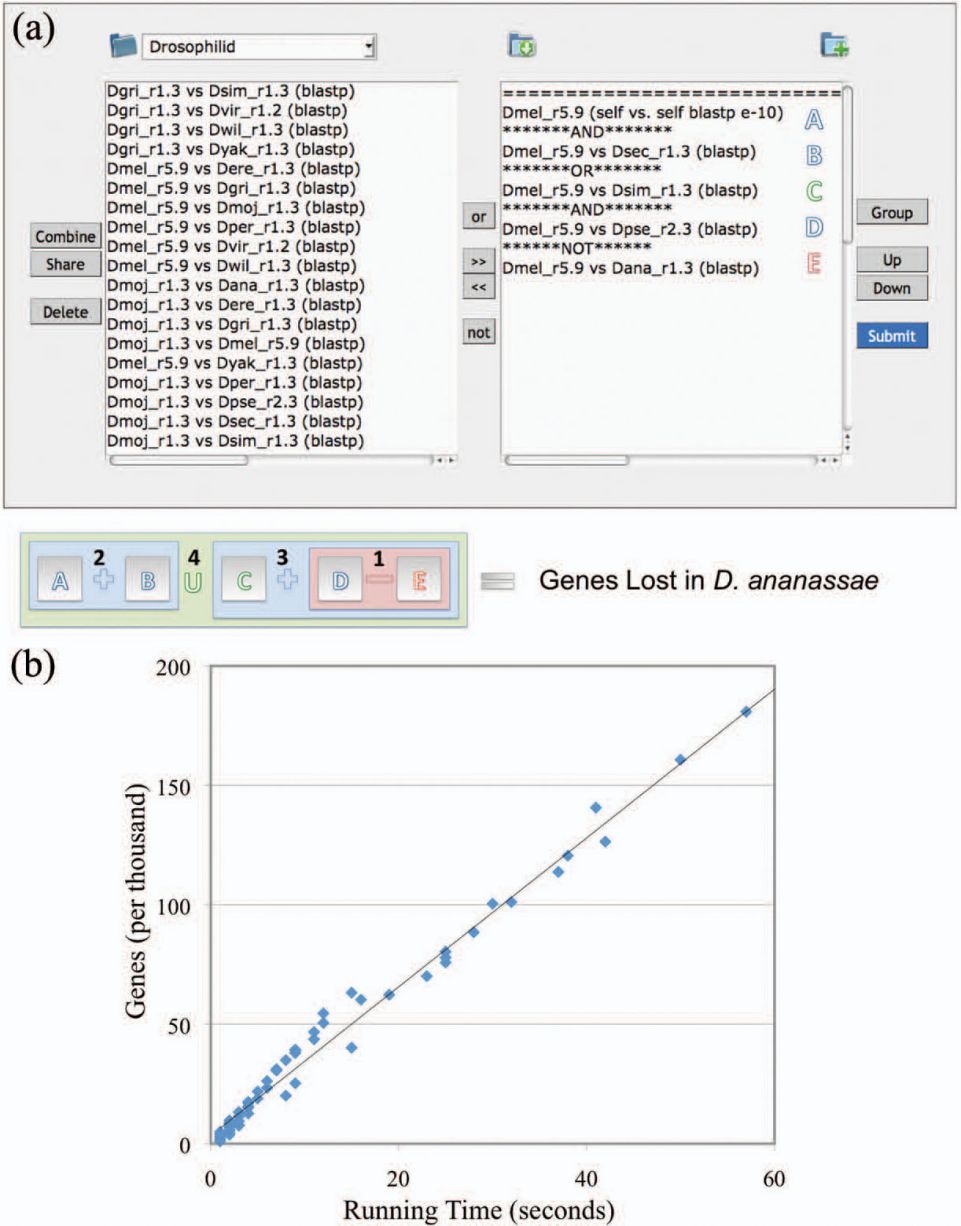
**Performing dataset merges with Booly**. **(a) **The right textbox depicts a list of datasets ready for Boolean operations. Numbered cartoon demonstrates the order of operations performed for this query. This query identifies genes lost in *D. ananassae *but are retained in the *melanogaster *subgroup and in the outgroup *D. pseudobscura *(see Additional file 2 [Figure S4a, Figure S4b]). Booly Precedence: 1) Group Selection Using Parenthesis. 2) NOT/Conjunction (-) Operation. 3) AND/Intersection (+) Operation. 4) OR/Union (U) Operation. Precedence for multiple instances of the same operator is determined by the order in which they appear in the query. **(b) **Approximate running time performing a Booly intersection with alias resolution. Y-axis contains the total number of combined identifiers (e.g. genes) for every dataset in a Booly merge. Plot shown represents 200,000 genes (10 intersections of datasets containing 20,000 genes apiece).

One can perform a combination of Boolean logic on multiple datasets by simply arranging datasets on our web interface in a manner akin to an algebra equation. We demonstrate the ability to perform powerful comparative analysis on the recently sequenced twelve *Drosophila *genomes to identify genes lost in one species of the *melanogaster *subgroup (Figure 2a, Additional file 1 [Suppl. Results], Additional file 2 [Figure S1], Additional file 2 [Table S1]).

**Figure 3:**
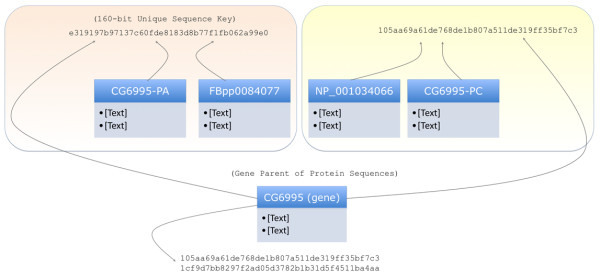
**Alias resolution of heterogeneous identifiers**. In the following example, the protein variants CG6995-PA and CG6995-PC have aliases FPpp00084077 and NP_001034066, respectively. When joined by a Boolean operation, the variants are kept separate due to having different unique sequence keys. However, if the proteins are joined with a list containing the gene parent (CG6995), the entire group is merged together if the gene has aliases that point to the protein variants.

Combining diverse datasets can be difficult when consideration must be made to map identifiers to a uniform nomenclature. A number of aliasing services exist which perform the task of alias resolution (DAVID, Synergizer, AliasServer, HGNC) [13-15], however many require pre-existing knowledge of an identifier's source before translation can be performed while others lack the flexibility to allow for aliases beyond just genes and proteins (e.g. aliases for drugs or ontology terms). To resolve these shortcomings, we have implemented our own streamlined form of alias resolution and demonstrate an approximate running time performing a Booly intersection with aliasing (Methods, Figure 2b, Figure 3).

### Privacy and Data Integrity

The Booly web application allows for users to create a secure personalized account for storage of datasets. In this manner, only the original owner of a data set will be able to view, modify, delete, and share their content. Once a data set is shared either publicly or to other individuals, permission is granted for the recipients to receive a copy of the data set, thereby preserving the original data set's integrity. The security of individual accounts is consistent with today's current web standards and will continually see improvements as the technology advances.

## Results

**Figure 4:**
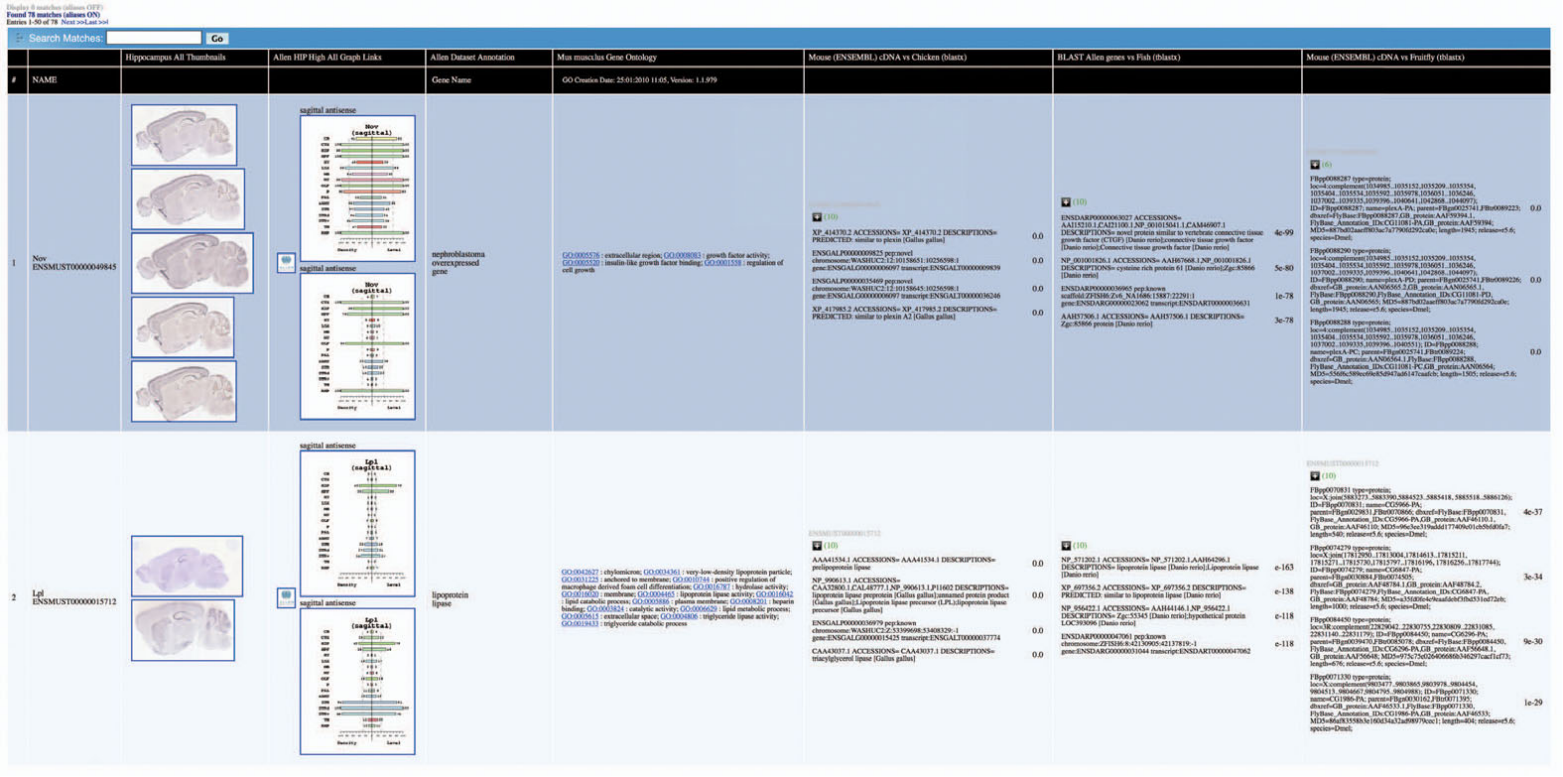
**A Booly query combining disparate datasets utilizing on-demand alias resolution**. Datasets merged in this Boolean query include annotation data, mouse brain expression summary graphs and *in-situ *thumbnails (Allen Mouse Brain Atlas), and BLAST summaries of mouse against the chicken, fish, and fruitfly. The results of this query can be accessed at: http://booly.ucsd.edu/hippocampus and a higher resolution version of the image can be downloaded at: http://booly.ucsd.edu/figures/Allen_results.jpg.

We illustrate the power of Booly's alias resolution while integrating multiple sources for the purpose of comparing mammalian and avian brain architecture. Our analysis began with a homebrew dataset we curated from the Allen Institute Brain Atlas for genes that are selectively expressed at high levels in the mouse hippocampus [16]. The next step was to integrate this dataset with mouse Gene Ontology and BLAST [17] hits of the mouse genome against other species such as the fish, chicken, and fruitfly. Unfortunately, while the Allen data and Gene Ontology had identifiers mapped to official mouse gene names, our BLAST data had identifiers mapped to Ensembl [18] sequence identifiers. Using our aliasing tool, we overcame this commonly encountered problem and seamlessly integrated these datasets together, which resulted in the identification of an enriched set of genes that are expressed in a region of the avian brain believed to correspond to the mammalian hippocampus (Figure 4, Additional file 1 [Suppl. Results], Additional file 2 [Figure S2]).

### Extensions and Applications

In addition to the core functionalities we have previously described, Booly can be extended further by creation of new applications. For example, we created an application that allows researchers to generate new BLAST datasets. Another application allows the user to switch "touch points" (identifiers used to map one piece of data to another) [1], which makes it possible to perform concatenated series of complex Boolean comparisons. An example of the utility of this tool is to integrate known *Drosophila melanogaster *genetic interaction networks with human diseases and existing uses of FDA approved drugs to develop a new approach to identify new potential uses for drugs, sometimes referred to as drug repurposing (Additional file 1 [Suppl. Results], Additional file 2 [Figure S3], Additional file 2 [Figure S4]).

Additionally, we are in the process of creating an Application Programming Interface (API) utilizing RESTful web services (http://booly.ucsd.edu/api), which will allow developers an easy way to both import and retrieve data within Booly.

## Discussion

The growing volume of biological and medical information deposited within disparate databases has created an organization and data integration dilemma within the research community. Furthermore, new data not configuring to pre-existing specialized databases must await creation of new dedicated inclusive databases. We have created a novel tool, Booly, as a web application that solves key problems impeding current data integration efforts. An important feature of this system is a real time alias translation system, which we used to successfully integrate datasets with heterogeneous identifiers between Ensembl, gene symbols and gene ontology. Secondly, we addressed the issue of the entry barrier by creating an easy to use contribution model for both developers and researchers. Users are able to easily add datasets by copying and pasting their spreadsheet tables or by utilizing applications designed to create new Booly datasets. Lastly, we showed how Booly could be used as an intermediate step in data mining and data integration through our implementation of the switching and chaining technique to change "touch points".

There are a myriad of other enabling applications for Booly. For example, as personalized genomes become available to the general population, Booly is poised to offer individuals space to house their biological and medical information such that it can also be used to compare with publicly available content in a safe and secure fashion. Booly is also a resource for developers to add content without the obstacle of creating an online storage facility or the troublesome nature of alias resolution. Booly thus offers a fundamentally new paradigm for storing, sharing, and integrating current and future health and biological content.

### Future Directions

Boolean modeling is a formal description of a broad array of biological phenomena, one notable example being gene regulation [19]. To this extent, many biological processes can be modeled by using Boolean Networks. Booly offers an important functionality for system level studies as it greatly facilitates integration of diverse datasets from multiple experimental sources, providing the first step in gathering data into a Boolean model. Further development of algorithms that apply networking or clustering of touch points within the groupings created by Booly could similarly lead to novel systems based hypotheses.

Booly offers a Uniform Resource Locator (URL) based web API, allowing developers to easily integrate their applications and datasets into Booly. In this manner, developers will be able to create their tool or database and use Booly as a repository for the tool's output. For example, an external database may allow users to directly download all of the results from a search and place them directly into the user's Booly account. The output generated from these tools, once placed inside Booly, will inherit all its functionality, including the ability to easily share the data, to perform Boolean logic comparisons with other data sets, and to resolve aliases.

An obvious concern for comprehensive databases, and thus for Booly, is the issue of scalability. That is, how will Booly deal with the exponential growth of data deposited into its systems? For example, as personalized genomes become a reality, as is currently being implemented in the 1000 Genomes Initiative [20], a means for an individual to store and explore this information will be highly desirable. We have created Booly in such a way that as the data grows, additional machines can be introduced in parallel into the system for load balancing and data partitioning without adversely affecting the Booly's efficiency (speed) and reliability (uptime).

A large component of Booly is the user contribution model as similarly applied to such online applications as Wikipedia and more relevantly, WikiGene [21]. However, a major concern is quality control of user-contributed data. Our plan to address this dilemma is to implement a community based review system for each dataset (similar to Amazon product ratings). In this manner, users will be able to search and add datasets based on "collective intelligence", a key element of Web 2.0 [22].

With most if not all data integration platforms, there is a concern that data can quickly grow out of date and require updating. The goal for Booly is to become a publically driven data repository reviewed and updated by its community. To aid the community in their efforts, we hope to implement a notification system such that when a new dataset is available, subscribers to the old dataset will be notified and allowed to upgrade or add the new dataset. In the meantime, we have created a forum message board so that contributors can disseminate update information to the community.

Finally, there is a growing movement in the life sciences to develop tools for semantic integration by way of the Resource Description Framework (RDF) model [11,23]. Semantic integration approaches involve establishing complex relationships between objects, which can then be used to classify them or extract novel information regarding their behaviours. The goal of Booly is more modest, to establish identity between objects and to use this information to integrate data in which distinct names refer to the same objects. We felt our initial challenge was to help researchers and developers get their data quickly onto the web and to address the identity problem directly. However, to aid in interoperability with other data integration efforts that utilize RDF and other semantic integration approaches, we plan to provide export of data into a structured model such as RDF. It is our hope that the streamlined but efficient and user friendly comparative tools offered by Booly attract a broad base of users who are confronted by the simple but vexing problem of integrating data from a diverse set of spread sheets. Such users once adept at using Booly would presumably be primed to expand their sphere of comparison by trying out new tools such as those offered by semantic integration approaches.

## Conclusions

Booly offers a new platform for the creation, storage, and integration of both personalized and public biological databases. As more applications are developed around the Booly platform, we anticipate these additions will further enhance the user experience. Booly presents a great opportunity to engage the research community in sharing data and adding combinatorial depth to potential queries. Such advances as offered by Booly should greatly aid researchers in formulating new questions that lead to novel discoveries in the laboratory.

## Availability and Requirements

Booly is freely accessible via most Javascript enabled web browsers at http://booly.ucsd.edu.

## Methods

### Design of Booly

Booly is comprised of the same components found in many current cloud computing web applications. These components can run on multiple servers and consist of a relational database that is load balanced and horizontally partitioned, custom scripts that access the database and perform Boolean operations, and a graphical user interface that can be accessed using a web browser. An overview of the Booly integration algorithm is shown in Figure 1. Further details of the Booly infrastructure and data integration components are described in Additional file 1 (Suppl. Methods).

### Booly Integration Algorithm

1. Dataset Ordering. Boolean operations are performed based on the order of precedence as described in Figure 2a.

2. Alias Hash Key Conversion. When aliasing is requested, all identifiers from every dataset (***D^1..n^***) are converted to a hash key from an in-house Alias lookup database. The hash key is derived by utilizing the Secure Hashing Algorithm 2 (SHA2) 160-bit digest of a fingerprint such as a gene sequence, chemical formula, URI, etc... (Figure 3, Additional file 1 [Suppl. Methods]). The hash key is unique to the fingerprint (avoiding collisions as is the problem with, e.g. numerical identifiers) and can convert any arbitrary length message into 40 hexadecimal characters. This makes the hash key ideal as a non-semantic identifier.

3. Identifiers Grouped Based on Aliases. Hash Keys as well as the original identifiers are grouped based on exact matches. Groups are then consolidated based on the criteria that identifiers are one and the same when the same hash key exists amongst all identifiers in question.

4. Consolidated Groups Undergo Boolean Operation. The first pair of datasets (***D^1^, D^2^***) based on Step 1 undergo the requested Boolean operation. The operation is performed iteratively until all matched aliases between the two datasets are exhausted.

5. Datasets Combined. The results of Step 4 are combined into a temporary dataset (***D^1,2^***). ***D^1,2 ^***is compared against ***D^3 ^***and steps 4-5 are repeated until a final dataset ***D^1..n ^***emerges.

## Authors' contributions

FFE carried out all *in situ *hybridization experiments to compare mammalian brain architecture and participated in the design of the study. HJK participated in the conception and design of the experiments. EB conceived of the study, participated in its design and coordination, and helped to write the manuscript. LHD conceived of the study, implemented Booly, and wrote the manuscript. All authors read and approved the final manuscript.

## Supplementary Material

Additional file 1 **Supplemental Results and Methods**. The supplemental results section contains further in-depth analysis of our findings as described here in the results section as well as additional examples on the use of Booly. The supplemental methods section provides further in-depth description of the components that make up the Booly platform. The file is a Microsoft Word formatted document. Click here for file

Additional file 2 **Supplemental Tables and Figures**. Additional tables and figures in a single Microsoft Word formatted document. Click here for file
