# Supplementary material for: Booly: a new data integration platform
Source: BMC Bioinformatics. 2010 Oct 13;11:513. doi: 10.1186/1471-2105-11-513 (PMC2970612; doi:10.1186/1471-2105-11-513)
Supplement: Additional file 1 — Supplemental Results and Methods. The supplemental results section contains further in-depth analysis of our findings as described here in the results section as well as additional examples on the use of Booly. The supplemental methods section provides further in-depth description of the components that make up the Booly platform. The file is a Microsoft Word formatted document. [file 1471-2105-11-513-S1.DOC]

# Supplemental Results

**Alias Resolution for Integrating Diverse Datasets**

A novel and important feature of Booly is its inherent ability to combine data from diverse sources into single comparative tables. One practical example that illustrates this enabling power of Booly is in helping to define sets of candidate genes that might be expressed in corresponding functional regions of the mammalian versus avian brains.

We were interested in testing the hypothesis that even though avian and mammalian brains appear superficially different in organization, that there are clear homologous regions in these brains as indicated by shared localized patterns of gene expression [1]. As a starting point, we took advantage of the genome-wide expression data in the mouse brain generously made available by the Allen Institute. We searched for genes expressed within well defined subdomains of the murine hippocampus such as the dentate gyrus and the CA1-CA3 subdivisions of Amon’s Horn since these discrete subdomains are defined and delimited by multiple gene expression patterns [2, 3]. As a test of the conserved brain structure hypothesis we could then ask whether the avian orthologs of these signature genes are similarly expressed in similar patterns in corresponding regions of the avian brain.

To identify well-conserved hippocampal gene markers for our studies we used the Booly’s ability to resolve aliases for identifiers of different datasets in our study of the vertebrate hippocampus. We searched the Allen Institute’s In Situ Brain Atlas of over 20,000 gene expression patterns for genes that are highly expressed in the mouse hippocampus and then constrained our list of candidates to those genes that exhibited high sequence conservation with genes in other vertebrate and invertebrate species including the chicken as a representative of birds and *Drosophila* as an invertebrate (Fig 3a.). Although the Allen Institute does an admirable job of mapping gene symbols to the identifiers of sequences, we encountered a number of examples where sequences were identified from different sources (mixtures of RefSeq identifiers and other NCBI accessions—most likely as a result of unavailable RefSeq curation). Some hippocampus specific genes were also identified in previous studies that used a different set of identifiers [3]. We therefore employed the Booly to perform the alias translations between the Allen data (mouse gene symbols) and data containing previous BLAST homologs of the mouse (Ensembl) to those of non-mammals (Ensembl). We were able to also integrate other datasets such as Gene Ontology avoiding the complication of having to conform to a singular identifier source.

Our data integration of the Allen Data with homologous BLAST datasets generated ~150 evolutionarily conserved candidate genes. We then reduced this list further to a set of 13 test genes by focusing our initial attention on genes expressed in highly localized patterns that encode transcription factors as well as those involved in restricted cell-cell signaling in the hippocampus (e.g., specifically in the CA1 region or the dentate gyrus). One clear prediction of the homology hypothesis (i.e., that there should be conserved patterns of gene expression in corresponding regions of the mammalian and avian brains) is that the chicken orthologues of mammalian hippocampal genes should at least be expressed, and presumably enriched, in regions of the bird brain that have been proposed to include the avian hippocampus based on functional studies. To test this hypothesis, we performed Real Time Quantitative Polymerase Chain Reaction (RTqPCR) analysis on RNA extracted from micro-dissected regions of the chick brain. The data provide a quantitative measure of relative gene expression levels in different regions of the brain, and are highly reliable as repeated RTqPCR runs produced highly concordant results as did analysis of RNA from brain regions dissected in independent experiments (Suppl. Fig. 2). We compared expression levels of these predicted avian hippocampal genes in the 1 day old chick hippocampus to that of other regions in the chick brain. We found approximately half of the genes tested showed at least a two fold elevation in gene expression in the hippocampus relative to that of neighboring regions in the chick brain. The Allen Institute database lists 286 genes (out of approximately 20,000) as being highly expressed in the hippocampal region/hippocampal formation in the mouse brain. This constitutes only 1.5% of the total genes tested in the mouse genome. In contrast, our hit rate was approximately 30-fold higher among our predicted set of avian hippocampal genes. We also validated our RTqPCR results in several cases by *in situ* hybridization and found that these genes were indeed expressed in a localized fashion in the proposed hippocampal region of the chick brain (F. Esteves et al., manuscript in preparation). Identifying a limited set of gene candidates to test experimentally in this encouraging pilot study was greatly facilitated through use of Booly and its ability to seamlessly resolve aliases.

**Data Integration for the General User**

Booly simplifies the database schema used to store datasets (Suppl. Fig. 5) so that the entry barrier for contributions has been dramatically reduced. General users can simply cut and paste their data from widely used formats such as Excel tables into Booly as new datasets. Developers can create more complex datasets through the use of HTML, particularly nested HTML tables as well as specialized data analysis tools (see below). Once datasets are created, they are stored within a user’s account and can be shared with others for distributed reuse.

Another method for users to create new datasets is through the use of applications that produce output placed directly into Booly. For example, given the broad utility of BLAST for sequence comparison, we incorporated this program such that users can create new BLAST datasets within Booly. This direct access to BLAST greatly increases the convenience and speed of performing searches such as for creating pairwise BLAST comparisons of all twelve *Drosophila* genomes. These Booly tables in turn can then be used efficiently for comparative analysis [4, 5] by performing a variety of consecutive intersections (AND) and a NOT operation to determine sets of genes that were lost at various nodes in the phylogenic tree. In one such analysis, we examined genes lost during evolution of *Drosophila ananassae* that were retained in the sister melanogaster subgroup comprised of *D. melanogaster, D. simulans, D. sechelia, D. yakuba*, and *D. errecta* and a neighboring out group, *D. pseudoobscura* (Suppl. Fig. 1a). Interestingly, the types of genes lost specifically in the *D*. *ananassae* lineage (73) fall into similar functional classes as those we and others have previously identified as being common classes for organism specific genes [4, 6] (Suppl. Fig. 1b, Suppl. Table 1). These genes include those involved in interaction with the outside world such as barrier forming chorion proteins (e.g., Cp18), and odorant binding (Or88a, Obp8a), defense (e.g., Tot family defensins), and reproductive signaling (OsC: pheromone binding).

**Booly as an Intermediate Platform for Touch Point Integration**

As described by Goble et al., “touch points” are targets of various efforts in data integration [7]. Booly employs a data model where identifiers (key—short text) are attached to every row of data (values can be text or html). Booly’s initial efforts for data integration involve the use of Boolean logic against the keys of each dataset. When two keys match (either directly or through alias translation), the values are brought together and the appropriate Boolean logic is performed. Our next goal was to demonstrate how Booly could be used as an intermediate step in extracting other touch points for further data integration. For this task, we first created a utility where combined datasets integrated by our Boolean logic functions can be saved as an entirely new dataset. We then added a functionality that allows for switching the identifier columns of the primary output tables, essentially changing the “touch point” to a new identifier (Suppl. Fig. 3a, Suppl. Fig. 4). Switching identifiers allows users to compare datasets that were originally not amenable to Boolean joining. This process, “switching and chaining”, which can be carried out in a concatenated fashion with multiple comparisons, allows for the creation of entirely new integrated datasets extending far beyond those that could be generated by standard Boolean comparisons constrained to datasets with common identifiers.

An example that illustrates the types of sophisticated analysis that can be accomplished using the switching and chaining technique is to identify a list of prescription drugs that could potentially be used to treat additional diseases outside of its known or intended target, a goal sometimes referred to as drug repurposing. One way to create a list of such candidate alternative drug uses is to link diseases into interaction networks (such networks often represent integrated biological processes such as signalling pathways or DNA repair) under the hypothesis that one might be able to use a drug treating one particular disease in the network for a second disease belonging in the same network (Fig. 3b). Since many diseases can be caused by mutations in genes or are related to such diseases, we first created a link between drugs, human diseases, and human disease genes. As human disease gene interaction data is not readily available, we identified homologs of human disease genes in the model organism *Drosophila melanogaster* and then utilized high quality genetic interaction data derived from the vast published literature available for this model system. In practical terms, we first linked the human disease to a gene (gathered from human genes known to have allelic variants for the particular disease-OMIM) and its prescribed drugs (FDA drug database), then we found the homolog of the human gene to that of the fruitfly, and finally we integrated genetic interaction networks found in the fruitfly (restricted to high quality interactions, ~1400 *D. melanogaster* genes). The entire process is summarized in Figure 4, which involves 3 instances of both switching and chaining of queries.

After performing this concatenated combinatorial operation, we retrieved a list of ≈ 50 genetic interactions that suggested potential alternative drug targets (<http://booly.ucsd.edu/drug-networks>). In one such example, the *forkhead* fly gene was shown to interact with *bkn* via a phenotypic enhancement. It’s closest human homologs were NP_036315-- linked to autoimmune diseases, and NP_005140--linked to hormone deficiencies, respectively. A particularly interesting drug, Cytomel, was retrieved for treating the general category of hormone deficiency. After closer inspection, it was found that Cytomel is used to treat cases of hypothyroidism. Hashimoto's thyroiditis, or chronic lymphocytic thyroiditis, is an autoimmune disease where the body's own T-cells attack the cells of the thyroid and is the most common form of hypothyroidism in the United States. Our study was able to reveal this particular connection between autoimmune disease and thyroid hormone deficiency. A question arising from this example is whether other drugs listed to treat various forms of hormone deficiency could be used to treat the other autoimmune disease, and vice versa, whether any of the drugs used to treat autoimmunity could be used to treat certain hormone deficiencies such as Hashimoto’s thyroiditis. Another example of a potential connection between genes that could have therapeutic implication is one between the multi-EGF domain Crb1 involved in stabilizing the adherens junction and another cell junction molecule DLG3 (<http://booly.ucsd.edu/drug-networks-2> ). Mutations in Crb1 cause Retinitis Pigmentosa (RP), a retinal degeneration disease, while disruption of DLG3 function causes mental retardation. No treatments are currently available to treat RP, however, patients with DLG3 mutations can be treated with anti-depression drugs such as Mirtazapine, Fluphenazine Hydrochloride, Buphenyl, or Prolixin Decanoate. Given the strong genetic interaction between the fly homologs of the human disease genes (crb ≈ Crb1 and DLG3 ≈ sdt) and the fact that they both play an important role in stabilizing cell-cell junctions one might wonder whether treatment of RP patients with drugs used to treat depression might have a positive effect. One could extract similar potential repurposing of drugs for the other drugs/diseases. To construct this particular list of candidate drugs for new diseases, we used only a small subset of interaction data in fruit flies and drug components. However, one could also perform additional queries of this kind based on other types of genetic interactions (e.g., in yeast, *C. elegans*, or mice) or using well validated protein-protein interaction data. This strategy offers a potentially useful alternative and complementary approach to existing attempts at drug repurposing based on categorizing disease states by virtue of shared gene expression profiles [8].

Our switching and chaining approach is only one example of how Booly can be used as an intermediate platform in data integration. The value fields can be extracted for touch points via other approaches and algorithms in a similar manner to how we extracted new identifiers. Coupled with an initial alias translation and Boolean logic functions, Booly offers core functionalities vital to future data integration efforts, which we anticipate will be further empowered as developers make use of its flexible simple format to create new functionalities that extend its utility.

# Supplemental Methods

**I. The Booly Infrastructure**

Specialized databases such as PUBMED or GenBank have been optimized in such a way that one can, for example, rapidly retrieve published references or sequence information. This specialization requires unique organization and entry methods to accommodate the differences in each database. Our goal has been to create an infrastructure such that any web based content can be stored inside our system without the overhead of more specialized databases; i.e., we want to be able to store PUBMED entries, GenBank sequence entries, images--all types of web-based data, using a singular database schema (Suppl. Fig. 5).

**Key to Value Relationship**

At the heart of our singular database schema is the key to value relationship. The “key”, or identifier, is simply a label for each row of data while the “value” is the actual row of data. Keys can be gene names, protein names, or any symbolic string (50 maximum). Values on the other hand, are data that do not have length constraints. Values can be any length of text, including HTML. The use of HTML allows one to create nested tables, multiple columns, hyperlinks, and numerous formatting options. The minimal requirements of a key to value relationship in any piece of data added makes Booly amenable to both diverse current and future data storage needs.

**User Data Management**

The most dynamic component of Booly is the ability of users to create their own content and store this information on the server. To keep track of each user’s data, we integrated an open source forum based login system (phpBB) into Booly. This feature offers not only a reliable account management system, but also a forum for communication with developers and other Booly members. Users can share datasets while also having access to any datasets made publicly available by other members. For example, users that have access to a list of *C. elegans* RNAi feeding libraries can easily submit their dataset to the public repository for others to use and perform Boolean queries against their list of *C. elegans* genes. If privacy is a concern, one can restrict sharing of datasets to colleagues through private email rather than by submitting the data to the open access public repository. These lists can then be added for comparison to the publicly available data from the users own local terminal.

A foremost concern in the construction of Booly is its potential to grow exponentially in size as more and more users store data. Although space concerns can be remedied by hardware upgrades, the speed at which user data is accessed can degrade as the database grows. To address this issue, a horizontal partitioning scheme is used to separate users into different groups. In this manner, the load can be spread across different locations (i.e. across different tables as well as different hardware).

**II. Data Integration**

An important question regarding such an open-ended database is, how will we integrate and compare information when we do not know its contents? Booly takes advantage of the key to value relationship by utilizing the keys to find commonalities between disparate datasets. If two keys are identical, Booly performs the appropriate Boolean logic. Furthermore, the usefulness of comparing keys is extended by the use of an integrated aliasing system, which detects the same gene or protein, essentially grouping aliases together under the same key. Finally, a method to export results and save them as entirely new datasets creates the possibility of higher levels of analysis within Booly.

**Integration Using Boolean Logic on Keys**

Booly integrates data between sets by utilizing the “keys” attached to each “value”. Boolean relationships between two sets include the union (or), intersection (and), and subtraction (not) operations. In Booly, Boolean queries are constructed dynamically as a user chooses datasets upon which to perform the Boolean operations. Datasets are evaluated row by row to find commonalities between each dataset compared. When keys match, rows of data are accordingly operated upon using Boolean logic. Boolean operations are resolved by a combination of database commands and an in-house developed implementation of Boolean logic. Finally, results are formatted for proper display on a web browser. We briefly describe the methodology and order of operations involved in submission of a query. A more complete description of database commands and in-house code is available upon request. A simple example of the use of such Boolean functionality is to generate mini databases for organisms with sequenced genomes that allow users to search for homologs of human genes causing disease (Table 2), as has been done previously for *Drosophila* genes in the Homophila database [9,10].

**The Query Interface**

The user constructs a Boolean operation by ordering datasets in an appropriate sequence and placing Boolean commands between each dataset (Fig 1, Fig. 2a). For example, the “OR” disjunction operation is constructed by adding the “or” command between two datasets, while the “NOT” negation operation requires addition of the “not” command between them. By default, the “AND” conjunction command is inferred if no Boolean operators are specified between two datasets. Therefore, adding two datasets to a list without any operators would result in output containing entries that exist in both datasets.

**Aliases**

As mentioned previously, identifier keys are compared against one another to perform Boolean operations between datasets. A daunting aspect of biological data is the abundance of different names for genes and proteins. For example, a gene can be referred to in published text using its gene symbol, while the same gene in a microarray experiment listed in tabular form is represented with an NCBI accession name. This presents a myriad of problems for indentifier based comparison operations. With the growing number of different aliases for each gene and its protein product, grouping different data sets together by the methods used in Booly seemed a daunting task.

There have been scattered bioinformatic attempts in dealing with biological aliases. Some are species specific, attempting to centralize all aliases for a particular species such as SGD for *Saccharomyces cerevisiae* [11], MGD for *Mus musculus* [12] and FlyBase for *Drosophila melanogaster* [13]. Other databases attempt to store aliases for all genes including gene transcripts as well as protein products such as NCBI Gene, and Gene Ontology [14]. There are also databases that are dedicated to providing only alias information such as AliasServer [15], and HGNC [16-18]. Although many of these databases have capable techniques to deal with aliases within their own databases, real time retrieval of such aliases for use in the Booly is not efficient or scalable. Furthermore, many alias translation services cannot easily decipher heterogeneous identifiers (AliasServer [15], HGNC [16-18], Synergizer [19]) or do not employ a singular alias output table such that all alias output sources must be selected to ensure complete coverage of every known alias (DAVID) [20].

Based on the underlying theory of Leibniz’s law, we have implemented our own streamlined solution to the alias problem (manuscript in preparation) and have integrated these results within Booly. In Leibniz’s law, if the properties of two identities, gene A and gene B, are the same, then gene A = gene B. In our implementation, one such property utilized is that of gene or protein sequence such that when two identifiers have the same sequence, they are considered aliases of each other.

Briefly, our approach is to generically simplify how aliases are stored. We are concerned with only two properties: a fingerprint (e.g. sequence, chemical nomenclature) and its associated name. Therefore, two identifiers having the exact same sequence are considered aliases. Unlike other alias solutions, we do not group aliases by derived resource (e.g. RefSeq or Swissprot, etc…), organism, nor do we attempt to build out aliases from a common locus of origin. Rather, we construct non-hierarchical unconnected subtrees of aliases where each subtree consists of a unique fingerprint, identified by a unique 160-bit hash key. By not grouping identifiers into separate derived sources, we can easily translate every heterogeneous identifier into its hash key fingerprint, allowing us to minimize complexity involved in forming and looking up multiple tables. Furthermore, the output of alias queries are all known hash keys for the requested identifiers which allow one to reduce the number of output sources to a single table, ensuring coverage of every known alias and its hash key fingerprints.

Creating unconnected subtrees of aliases also allows context specificity. For example, if we build out aliases for a gene to include protein variants, we can create a one-to-many relationship with a gene to its protein products. However, when comparing only proteins to each other, variant relationships are maintained because the protein aliases are in a different subtree from the gene alias tree (Fig. 3).

When one performs a Boolean comparison, the identifiers in each list are resolved for aliases and grouped together by the database. Our programs retrieve the alias groups and performs the Boolean logic accordingly. Currently, we have created aliases for all the sequences in the non-redundant (nr) database from GenBank, all sequences from Ensembl, as well as sequence in the Gene Ontology Database totalling over 30 million unique alias-sequence combinations.

**Exporting Results**

Another important function that we have incorporated into Booly is the ability to export results once a Boolean query has been performed. Two options exist when exporting: 1) a local save (as an xml file or html file) on a user’s personal computer, or 2) a remote save back onto Booly as an entirely new data set. The latter ability to create a new data set within Booly opens up new avenues of data integration. We briefly describe two applications, switching keys and keyword filtering, which both take advantage of exporting results as a new Booly dataset.

**Switching Keys**

The “key” in a key-value relationship is significant in Booly since it is the key that is used when comparing datasets against one another using Boolean logic. However, in some instances, the values may actually contain identifiers within them that could be used as keys for further Boolean operations (Suppl. Fig. 3a). By allowing users to switch keys during the export of Boolean results, one can chain together datasets and rename keys with identifiers derived from text within the values. This creates the possibility of integrating data within values and not just the keys. The powerful combination of switching keys and chaining together concatenated series of Boolean queries allows users to make sophisticated links between otherwise unconnected datasets.

**Keyword Filter**

Another feature to increase the value of exporting results of a Booly merge is the ability to filter out those results based on a keyword. An example of this usage is the ability to search for results that have gene ontology involved in immune response after a dataset of gene ontology has been merged with gene expression data. Users can search for key words and then export these results as a new Booly dataset.

**Further Integration Using External Applications**

An important aspect of Booly is the ability for users to store content such as output from a computer program. For example, we have developed a tool that allows users to perform BLAST comparisons and store its results directly inside Booly. The output of the job is tailored specifically for Booly and stored in the user’s account, allowing the user to retrieve and compare other datasets to the newly created BLAST dataset. We plan to provide a variety of additional plugin modules in the form of a Web API for developers in the near future. By allowing applications to directly submit output into Booly for data storage, users of the application would receive integration of their generated data with other applications as well as the ability to perform Boolean operations and alias resolution.

**Supplemental References**

1. Jarvis ED, Gunturkun O, Bruce L, Csillag A, Karten H, Kuenzel W, Medina L, Paxinos G, Perkel DJ, Shimizu T *et al*: **Avian brains and a new understanding of vertebrate brain evolution**. *Nat Rev Neurosci* 2005, **6**(2):151-159.

2. Lein ES, Hawrylycz MJ, Ao N, Ayres M, Bensinger A, Bernard A, Boe AF, Boguski MS, Brockway KS, Byrnes EJ *et al*: **Genome-wide atlas of gene expression in the adult mouse brain**. *Nature* 2007, **445**(7124):168-176.

3. Lein ES, Zhao X, Gage FH: **Defining a molecular atlas of the hippocampus using DNA microarrays and high-throughput in situ hybridization**. *J Neurosci* 2004, **24**(15):3879-3889.

4. Clark AG, Eisen MB, Smith DR, Bergman CM, Oliver B, Markow TA, Kaufman TC, Kellis M, Gelbart W, Iyer VN *et al*: **Evolution of genes and genomes on the Drosophila phylogeny**. *Nature* 2007, **450**(7167):203-218.

5. Stark A, Lin MF, Kheradpour P, Pedersen JS, Parts L, Carlson JW, Crosby MA, Rasmussen MD, Roy S, Deoras AN *et al*: **Discovery of functional elements in 12 Drosophila genomes using evolutionary signatures**. *Nature* 2007, **450**(7167):219-232.

6. Reiter LT, Do LH, Fischer MS, Hong NA, Bier E: **Accentuate the negative: proteome comparisons using the negative proteome database**. *Fly (Austin)* 2007, **1**(3):164-171.

7. Goble C, Stevens R: **State of the nation in data integration for bioinformatics**. *J Biomed Inform* 2008, **41**(5):687-693.

8. Hu G, Agarwal P: **Human disease-drug network based on genomic expression profiles**. *PLoS One* 2009, **4**(8):e6536.

9. Chien S, Reiter LT, Bier E, Gribskov M: **Homophila: human disease gene cognates in Drosophila**. *Nucleic Acids Res* 2002, **30**(1):149-151.

10. Reiter LT, Potocki L, Chien S, Gribskov M, Bier E: **A systematic analysis of human disease-associated gene sequences in Drosophila melanogaster**. *Genome Res* 2001, **11**(6):1114-1125.

11. Cherry JM, Adler C, Ball C, Chervitz SA, Dwight SS, Hester ET, Jia Y, Juvik G, Roe T, Schroeder M *et al*: **SGD: Saccharomyces Genome Database**. *Nucleic Acids Res* 1998, **26**(1):73-79.

12. Blake JA, Eppig JT, Richardson JE, Davisson MT: **The Mouse Genome Database (MGD): a community resource. Status and enhancements. The Mouse Genome Informatics Group**. *Nucleic Acids Res* 1998, **26**(1):130-137.

13. Gelbart WM, Crosby M, Matthews B, Rindone WP, Chillemi J, Russo Twombly S, Emmert D, Ashburner M, Drysdale RA, Whitfield E *et al*: **FlyBase: the Drosophila database. The Flybase Consortium**. *Nucleic Acids Res* 1996, **24**(1):53-56.

14. Ashburner M, Ball CA, Blake JA, Botstein D, Butler H, Cherry JM, Davis AP, Dolinski K, Dwight SS, Eppig JT *et al*: **Gene ontology: tool for the unification of biology. The Gene Ontology Consortium**. *Nat Genet* 2000, **25**(1):25-29.

15. Iragne F, Barre A, Goffard N, De Daruvar A: **AliasServer: a web server to handle multiple aliases used to refer to proteins**. *Bioinformatics* 2004, **20**(14):2331-2332.

16. Eyre TA, Ducluzeau F, Sneddon TP, Povey S, Bruford EA, Lush MJ: **The HUGO Gene Nomenclature Database, 2006 updates**. *Nucleic Acids Res* 2006, **34**(Database issue):D319-321.

17. Wain HM, Lush M, Ducluzeau F, Povey S: **Genew: the human gene nomenclature database**. *Nucleic Acids Res* 2002, **30**(1):169-171.

18. Wain HM, Lush MJ, Ducluzeau F, Khodiyar VK, Povey S: **Genew: the Human Gene Nomenclature Database, 2004 updates**. *Nucleic Acids Res* 2004, **32**(Database issue):D255-257.

19. Berriz GF, Roth FP: **The Synergizer service for translating gene, protein and other biological identifiers**. *Bioinformatics* 2008, **24**(19):2272-2273.

20. Huang da W, Sherman BT, Stephens R, Baseler MW, Lane HC, Lempicki RA: **DAVID gene ID conversion tool**. *Bioinformation* 2008, **2**(10):428-430.
