# Supplementary material for: Booly: a new data integration platform
Source: BMC Bioinformatics. 2010 Oct 13;11:513. doi: 10.1186/1471-2105-11-513 (PMC2970612; doi:10.1186/1471-2105-11-513)
Supplement: Additional file 2 — Supplemental Tables and Figures. Additional tables and figures in a single Microsoft Word formatted document. [file 1471-2105-11-513-S2.DOC]

**Supplemental Tables and Figures**

**Supplemental Figure 1. (a) Twelve *Drosophila* genomes.** *Drosophila* genomes that have been sequenced and an associated divergence timeline (http://rana.lbl.gov/drosophila). We subtracted genes of *D. ananassae* (red) from the subset of genes found in the genomes of the *melanogaster* subgroub and the outgroup *D. pseudoobscura* (blue). **(b) Genes lost selectively in *D. Ananassae*.** We identified over 73 genes that were lost during evolution of the *Drosophila ananassae lineage* that were retained in the sister melanogaster subgroup comprised of *D. melanogaster, D. simulans, D. sechelia, D. yakuba*, and *D. errecta* and in the outgroup *D. pseudoobscura*. Annotated lost genes fall into the same major functional classes as those that are found to be enriched among species-specific genes. The results of this query can be accessed at: http://booly.ucsd.edu/dana-lost.

(a)

(b)

**Supplemental Figure 2. RT-PCR analysis of genes predicted to be enriched in the avian hippocampus.** Relative fold change of selected genes in the Hippocampus and other areas of the Chick Brain, based on two or more individual independent experiments, which were highly concordant. Housekeeping genes GAPDH and actin were used as controls for normalization as described in the Methods section.


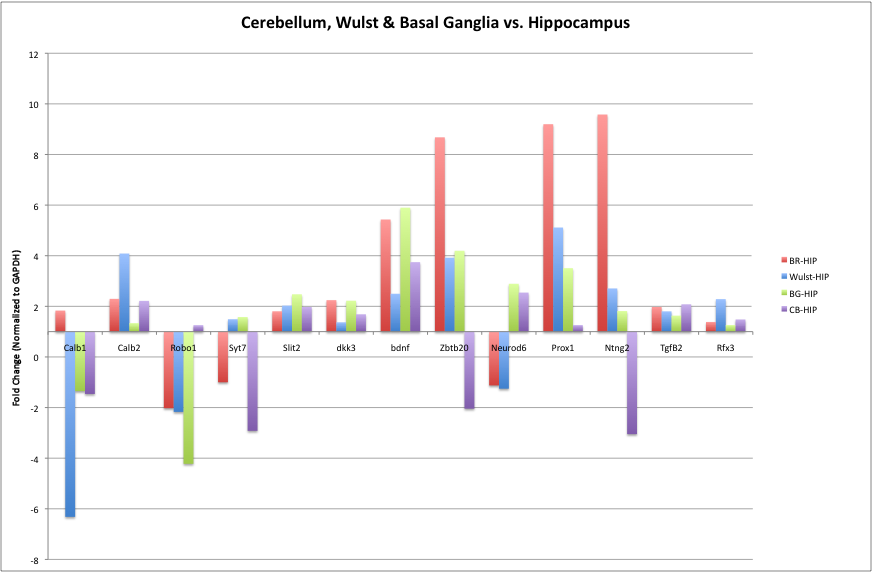


**Supplemental Figure 3. (a) Exporting Results and Switching Keys.** Example of exporting and saving as a new dataset inside Booly. A new key is assigned for each row by taking an identifier within the “value” field. **(b) Linking Drugs to Interaction Networks.** An example of a complex, chained Boolean comparison is the identification of new diseases that might be treated by FDA approved drugs currently used to treat a different disease. The idea is to first link a list of FDA approved drugs to diseases they can treat, then to associate genes with these diseases based on mutations in these genes causing phenotypes similar to the diseases treated by drugs, then linking these human disease genes to homologous genes in the fruit fly, then to broaden this list of genes to those interacting genetically with mutations in the fly gene homolog, then to ask whether any of the interacting fly genes have human homologs that also lead to disease, and finally to ask whether these potentially related human diseases might also be treatable with drugs used for the first disease, and vice-versa. The results of this query can be accessed at: <http://booly.ucsd.edu/drug-networks>.


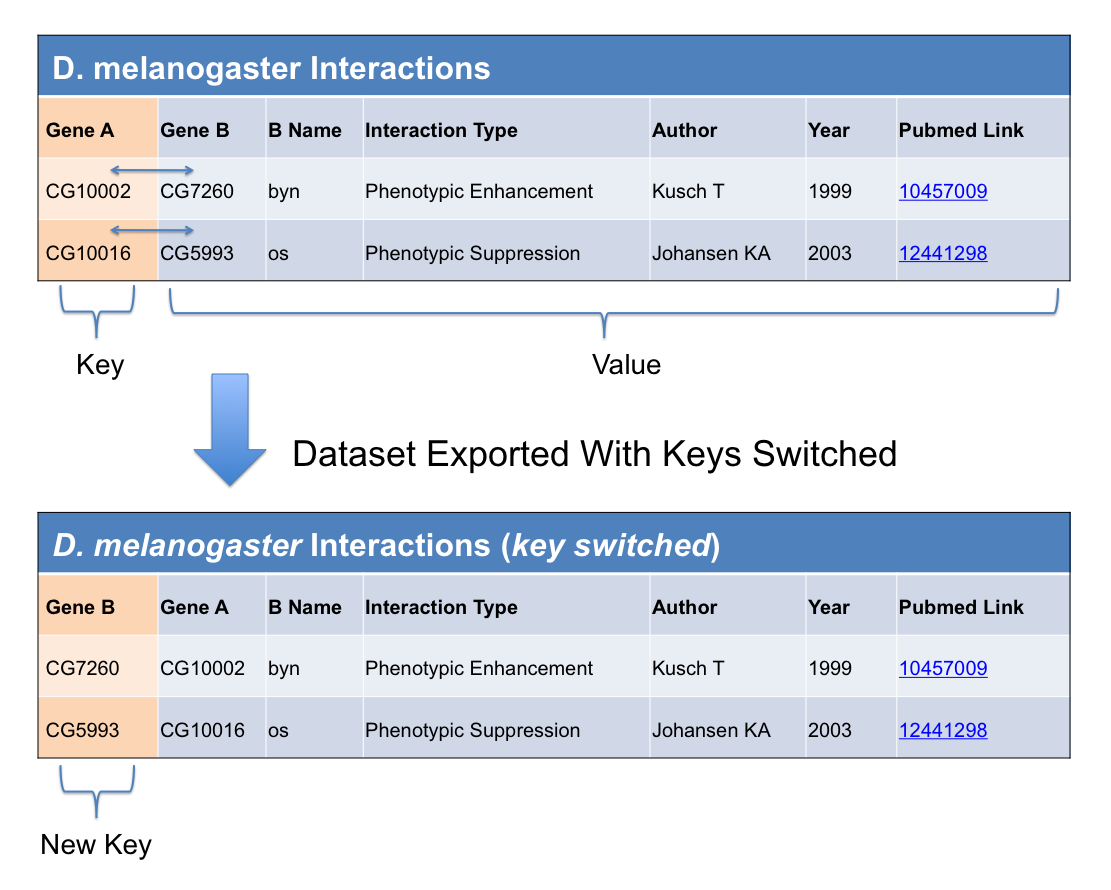


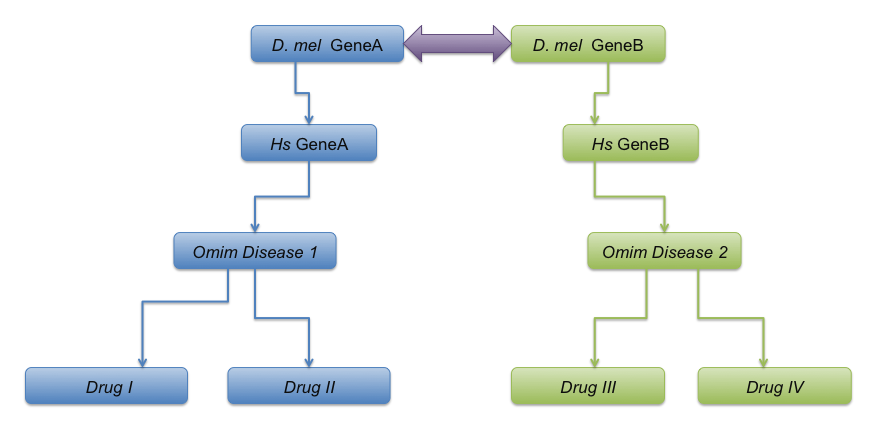


(b)

(a)

**Supplemental Figure 4. Switching Keys and Chaining Boolean Queries**. An example of switching “touch-points” so that two separate diseases and their associated drugs can be integrated within an interaction network found in *D. melanogaster* (*fkh* and *bkn*).


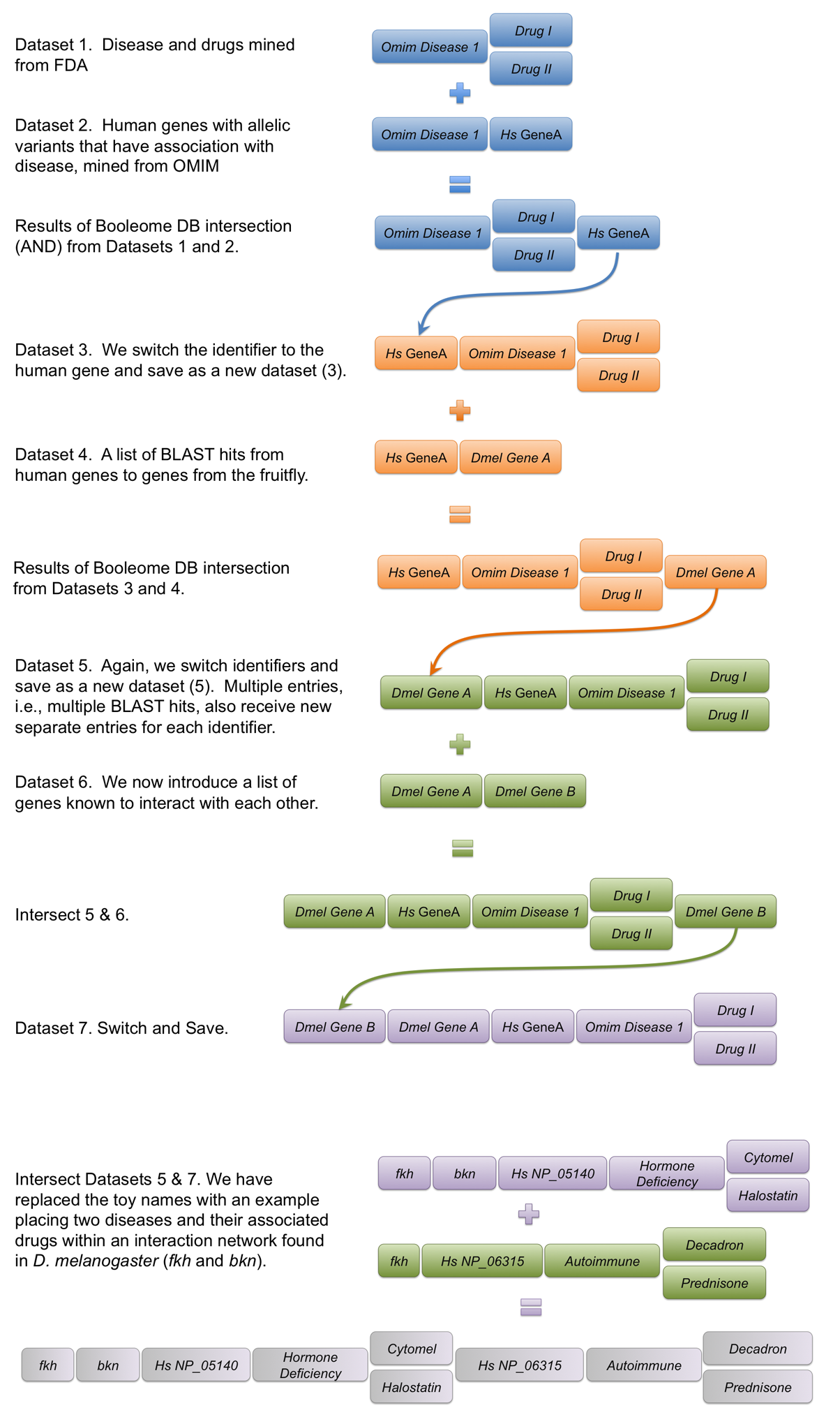


**Supplemental Figure 5. Database Schema for Booly.** Booly is an account based web tool which utilizes a relational MySQL database and custom scripts to perform Boolean merges between different datasets. The “Dataset” table consists of similarly structured tables horizontally partitioned across multiple servers (d_location). Each row of data contains a key, value pair. The key is the identifier for the value (text or html). Booly has integrated aliasing to group the same genes or proteins together.


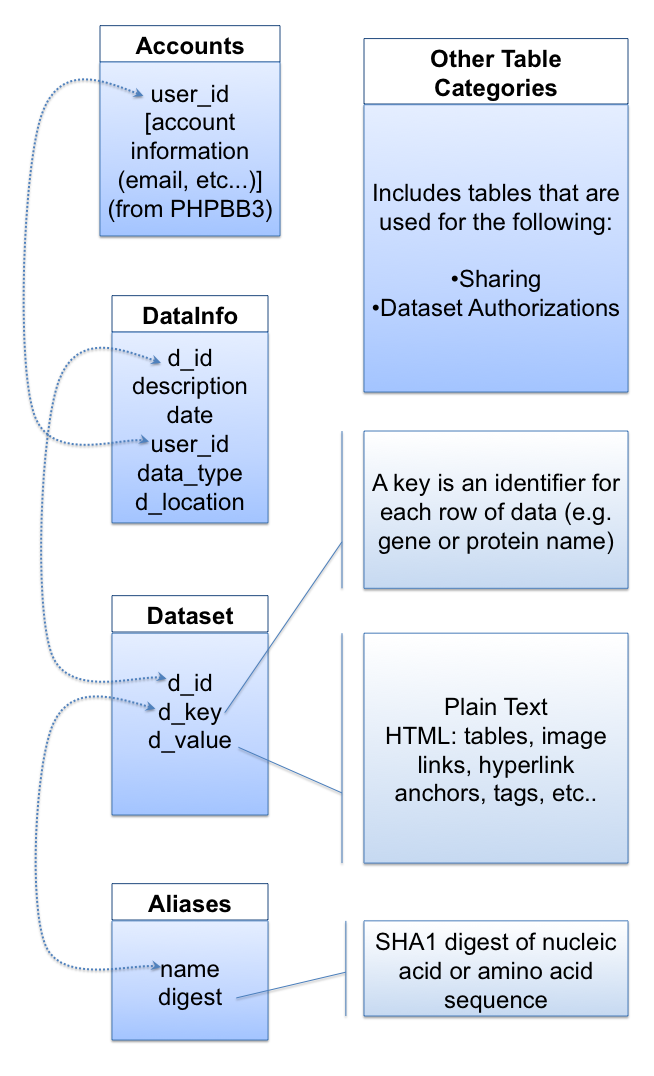


**Supplemental Table 1. Genes lost selectively in *D. Ananassae*.** We identified over 73 genes that were lost during evolution of the *Drosophila ananassae lineage* that were retained in the sister melanogaster subgroup comprised of *D. melanogaster, D. simulans, D. sechelia, D. yakuba*, and *D. errecta* and in the outgroup *D. pseudoobscura*.

| **Function** | **D. mel Gene** | **D. mel CG** | **D. mel Name** | **D. mel Gene Ontology** | ***D. mel* Protein Length** |
| --- | --- | --- | --- | --- | --- |
|  |  |  |  |  |  |
| **Defense** |  |  |  |  |  |
|  | FBgn0044811 | CG31691 | **TotF** | humoral defense mechanism (sensu Protostomia) | 125 |
|  | FBgn0031701 | CG14027 | **TotM** | humoral defense mechanism (sensu Protostomia) | 131 |
|  | FBgn0044810 | CG31193 | **TotX** | humoral defense mechanism (sensu Protostomia) | 142 |
|  | FBgn0053117 | CG33117 | **Victoria** | extracellular, "humoral defense mechanism (sensu Protostomia)" | 134 |
|  | FBgn0004240 | CG12763 | **Dpt** | extracellular, "antibacterial humoral response (sensu Protostomia)", "defense response to bacteria", "innate immune response", "NOT defense response to Gram-negative bacteria" | 106 |
| **Barrier Formation** | |  |  |  |  |
|  | FBgn0000357 | CG6517 | **Cp18** | structural constituent of chorion (sensu Insecta), "insect chorion formation", "chorion" | 172 |
|  | FBgn0041252 | CG15573 | **Femcoat** | structural constituent of chorion (sensu Insecta), "cytoplasm", "insect chorion formation" | 201 |
| **Chemosensation** | |  |  |  |  |
|  | FBgn0041232 | CG32395 | **Gr65a** | taste receptor activity | 408 |
|  | FBgn0038203 | CG14360 | **Or88a** | olfactory receptor activity, "odorant binding", "perception of smell", "NOT integral to membrane" | 401 |
|  | FBgn0034509 | CG13421 | **Obp57c** | odorant binding, "transport", "cellular_component unknown" | 149 |
|  | FBgn0030103 | CG12665 | **Obp8a** | odorant binding, "transport" | 163 |
| **Reproduction** | |  |  |  |  |
|  | FBgn0010401 | CG3250 | **Os-C** | pheromone binding | 131 |
|  | FBgn0000246 | CG17604 | **c(3)G** | synaptonemal complex, "structural constituent of cytoskeleton", "protein targeting", "cytoskeleton organization and biogenesis", "mitosis", "meiotic recombination", "microtubule binding" | 744 |
| **Metabolism** |  |  |  |  |  |
|  | FBgn0025809 | CG8962 | **Paf-AHalpha** | 1-alkyl-2-acetylglycerophosphocholine esterase activity, "phospholipid metabolism" | 225 |
|  | FBgn0044051 | CG14173 | **Ilp1** | insulin receptor binding, "hormone activity", "extracellular", "physiological process" | 154 |
| **Transcription** | |  |  |  |  |
|  | FBgn0033010 | CG3136 | **Atf6** | DNA binding, "nucleus", "regulation of transcription, DNA-dependent", "protein homodimerization activity" | 741 |
|  | FBgn0033459 | CG12744 | **CG12744** | nucleic acid binding, "nucleus", "zinc ion binding" | 160 |
|  | FBgn0037183 | CG14451 | **CG14451** | nucleic acid binding, "nucleus", "zinc ion binding" | 264 |
| **Translation** |  |  |  |  |  |
|  | FBgn0039739 | CG15527 | **RpS28a** | nucleic acid binding, "structural constituent of ribosome", "cytosolic small ribosomal subunit (sensu Eukarya)", "protein biosynthesis" | 64 |
|  | FBgn0011824 | CG4038 | **CG4038** | small nucleolar ribonucleoprotein complex, "rRNA processing", "35S primary transcript processing", "ribosome biogenesis", "rRNA binding" | 237 |
| **Proteolysis** |  |  |  |  |  |
|  | FBgn0033875 | CG6357 | **CG6357** | cysteine-type endopeptidase activity, NOT cathepsin L activity | 439 |
|  | FBgn0051704 | CG31704 | **CG31704** | serine-type endopeptidase inhibitor activity, "proteolysis and peptidolysis" | 68 |

**Supplemental Table 2. Homologs of human diseased genes**. This table illustrates the simple nature in which we are able to create new databases, rivaling previously created databases such as *Homophila* (<http://superfly.ucsd.edu/homophila>).

| **Species** | **Proteins** | **% Prots Hit to OMIM (e-03)** | **Link** |
| --- | --- | --- | --- |
| *Pan troglodytes* | 32852 | 52.1% | <http://booly.ucsd.edu/omim/chimp> |
| *Mus musculus* | 53815 | 48.2% | <http://booly.ucsd.edu/omim/mouse> |
| *Gallus gallus* | 22186 | 51.5% | <http://booly.ucsd.edu/omim/chick> |
| *Loxodonta_africana* | 15717 | 49.5% | <http://booly.ucsd.edu/omim/elephant> |
| *Danio rerio* | 36065 | 59.8% | <http://booly.ucsd.edu/omim/zebrafish> |
| *D. melanogaster* | 18498 | 45.1% | <http://booly.ucsd.edu/omim/fruitfly> |
| *C. elegans* | 23258 | 51.5% | <http://booly.ucsd.edu/omim/worm> |
| *S. cerevisiae* | 11081 | 24.4% | <http://booly.ucsd.edu/omim/yeast> |
| *E coli K12* | 4243 | 14.6% | <http://booly.ucsd.edu/omim/ecoli> |
